# MDMA targets miR-124/MEKK3 via MALAT1 to promote Parkinson’s disease progression

**DOI:** 10.1007/s11033-023-08775-w

**Published:** 2023-09-09

**Authors:** Xin Geng, Shipeng Li, Jinghui Li, Renli Qi, Lianmei Zhong, Hualin Yu

**Affiliations:** 1 The Second Department of Neurosurgery, The First Affiliated Hospital of Kunming Medical University, Kunming, 650032 Yunnan China; 2 Yunnan Provincial Clinical Research Center for Neurological Disease, Kunming, 650032 Yunnan China

**Keywords:** Parkinson’s disease, MDMA, MEKK3, MALAT1, miR-124, Neuronal damage

## Abstract

**Background:**

Parkinson’s disease (PD) is a well-known neurodegenerative disease that is usually caused by the progressive loss of dopamine neurons and the formation of Lewy vesicles. 3,4-Methylenedioxymethamphetamine (MDMA) has been reported to cause damage to human substantia nigra neurons and an increased risk of PD, but the exact molecular mechanisms need further investigation.

**Methods:**

MPTP- and MPP+-induced PD cells and animal models were treated with Nissl staining to assess neuronal damage in the substantia nigra (SN) area; immunohistochemistry to detect TH expression in the SN; TUNEL staining to detect apoptosis in the SN area; Western blotting to detect the inflammatory factors NF-κB, TNF-α, IL-6 and mitogen-activated protein kinase kinase kinase 3 (MEKK3); Griess assay for NO; RT‒qPCR for metastasis-associated lung adenocarcinoma transcript 1 (MALAT1) and miR-124 expression; Cell proliferation was assessed by CCK-8. Dual luciferase reporter genes were used to verify targeting relationships.

**Results:**

MDMA promoted MALAT1 expression, and knockdown of MALAT1 alleviated the MDMA-induced inhibition of SH-SY5Y cell proliferation, inflammation, NO release, SN neuronal injury, and TH expression inhibition. Both inhibition of miR-124 and overexpression of MEKK3 reversed the neuroprotective effects exhibited by knockdown of MALAT1.

**Conclusion:**

MDMA promotes MALAT1 expression and inhibits the targeted downregulation of MEKK3 by miR-124, resulting in upregulation of the expression of MEKK3 and finally jointly promoting PD progression.

**Supplementary Information:**

The online version contains supplementary material available at 10.1007/s11033-023-08775-w.

## Introduction

Parkinson’s disease (PD) is a well-known neurodegenerative disease that is usually caused by the progressive loss of dopamine neurons in the SN as well as the formation of Lewy vesicles [1, 2]. As an extrapyramidal disease, once suffering from Parkinson’s disease, the patient’s body will gradually stiffen and tremble involuntarily, as well as experience nonmotor symptoms, including olfactory dysfunction, cognitive impairment, confusion, apathy, mood disorders, and autonomic dysfunction (e.g., urinary and bladder disorders and constipation), which, once manifested, tend to persist and increase the care burden; psychiatric symptoms can even surpass motor symptoms as the primary factor affecting patients’ quality of life and survival [1, 3, 4]. PD patients can be found all over the world, but the disease mostly affects elderly individuals, with a prevalence of approximately 5.5% in people over the age of 60 [5]. Current treatments for PD are still very limited, and the chances of a patient’s full recovery are slim. However, medical research is constantly progressing, and it is worth actively pursuing new and reliable medical treatments for the disease. This work is also of great significance.

3,4-Methylenedioxymethamphetamine(MDMA), commonly known as ecstasy, is a psychostimulant that acts directly on the central nervous system, causing a decrease in the levels of striatal dopamine and its major metabolites [6, 7]. Rumpf JJ [8] et al. showed that methamphetamine abuse can lead to damage to nigrostriatal neurons and an increased risk of PD in humans; it has also been shown that MDMA administration in nonhuman primates not only damages serotonergic terminals but also dopaminergic neurons and enhances the neurotoxic effects of 1-methyl-4-phenyl-1,2,3,6-tetrahydropyridine (MPTP) [6]. However, the mechanism of the role of MDMA in PD pathogenesis is still unclear. Therefore, this research will provide a new breakthrough point for the study of the disease.

Reviewing a large number of studies and clinical evidence, we know that noncoding RNAs (ncRNAs) are highly expressed in the brain, and researchers suggest that they have multiple neuromodulatory functions. Numerous studies have also shown that the expression of long noncoding RNAs (lncRNAs) in the brains of patients with Parkinson’s disease presents a clear upregulation trend, and we are further convinced that they may have unique regulatory functions in neurological diseases. Among them, metastasis-associated lung adenocarcinoma transcript 1 (MALAT1), also known as nuclear-enriched abundant transcript 2 (NEAT2), has been shown to be aberrantly expressed in PD patients [9]. In addition, MALAT1 was also found to promote PD pathogenesis by inducing PD in cells and animal models [10, 11]. However, whether MDMA regulates PD pathogenesis through MALAT1 needs further study.

It is well known that noncoding small RNAs include a variety of RNA molecules, including miRNAs and siRNAs. Among them, miRNAs are relatively small in molecular weight and are 18–23 nucleotides in length. They can silence or degrade mRNA and then metabolize and degrade mRNA in biological processes regulating cell death. Among them, miR-124 is abundant in the brain and has multiple functions, including neurogenesis and neurotransmission [12]. According to a large number of studies, the expression of miR-124 is downregulated in the brain tissue of PD patients, induced PD animals and cell models [13, 14], and overexpression of miR-124 alleviates the pathogenesis of PD [14]. Hence, the level of miR-124 in plasma was considered a potential diagnostic marker for PD [12]. Moreover, mitogen-activated protein kinase kinase kinase 3 (MEKK3) is a target gene of miR-124, and MEKK3 can be regulated in PD to suppress neuroinflammation [15]. However, it is unclear whether miR-124/MEKK3 is involved in MDMA-mediated regulation of PD pathology. Therefore, this will be the focus of our research.

## Materials and methods

### Cell culture and transfection

Human SH-SY5Y cells were purchased from Otwo Biotechnology Co., Ltd. (Shenzhen, China) and were grown and passaged in DMEM containing 10% fetal bovine serum and 0.1% penicillin‒streptomycin. The medium was changed every 3–4 d, and the cells were collected and dispersed at 65-75% confluence. After resuscitation, the cells were transferred to 3 ~ 5 generations for the experiment. According to the literature [16, 17]. The negative control, si-MALAT1, miR-124 inhibitor, and OE-MEKK3 were synthesized by RiboBio (China) and transfected into SH-SY5Y cells by Lipofectamine 2000. Then, SH-SY5Y cells were exposed to 1 mM MPP + for 24 h to construct a PD cell model.

### CCK-8 experiment

SH-SY5Y cells were inoculated into 96-well plates, and the cells were transfected for 24 h using treatment with 1 mM MPP + for 24 h. After adding CCK-8 solution (Dojindo, Japan) to the sample and modulating the enzyme label, the absorbance was measured on a spectrophotometer at an absorbance of 450 nm.

### RT‒qPCR

A TRIzol RNA extraction kit (Thermo Fisher Scientific, USA) was used to extract total RNA from the collected cells and tissues. Then, the first strand of cDNA was assembled using the total RNA of the sample as a template, and the cDNA obtained was used as a template for qPCR amplification. The subsequent PCR process was completed using cDNA, as well as GAPDH and U6 as the internal reference, according to the instructions of the SYBR Green qPCR kit (Takara Biotechnology Co., Ltd, China). Then, the expression levels of cells and tissues were calculated using the 2^–ΔΔCT^ method. The primer sequences of MALAT1 F/MALAT1 R, miR-124 F/miR-124R, U6 F/U6 R and GAPDH/GAPDH R are displayed in Table 1.

**Table 1:** The sequences of primers for RT‒qPCR

Genes	Primer	Sequences
Mouse MALAT1	Forward	5ʹ-TTGGACTTGAGCTGAGGTGCTT − 3ʹ
	Reverse	5ʹ-CACCAAACTGGCTTCGGGAC-3ʹ
Homo sapiens MALAT1	Forward	5ʹ- GCAGCCCGAGACTTCTGTAA-3ʹ
Mouse GAPDH	Reverse	5ʹ- GTTATGCCTGGTTAGGTATGAG-3ʹ
	Forward	5ʹ- CACCATCTTCCAGGAGCGAGAC − 3ʹ
Homo sapiens GAPDH	Reverse	5ʹ- AGCCCTTCCACAATGCCAAA − 3ʹ
	Forward	5ʹ- AATCCCATCACCATCTTCCAG − 3ʹ
miR-124	Reverse	5ʹ- GAGTCCTTCCACGATACCAAAG − 3ʹ
	Forward	5ʹ- CGTAAGGCACGCGGTGAA − 3ʹ
U6	Reverse	5ʹ- AGTGCAGGGTCCGAGGTATT-3ʹ
	Forward	5ʹ- CTCGCTTCGGCAGCCACA − 3ʹ
	Reverse	5ʹ- AACGCTTCACGAATTTGCGT − 3ʹ

### Western blotting

The total protein of each group was extracted and lysed, as well as subjected to mass determination. Then, electrophoresis separation and mold transfer were performed. Then, the fixed membrane was incubated with primary antibodies (Abcam, UK), including NF-κB, TNF-α, IL-6, and MEKK3 (all 1:1000), and horseradish peroxidase (HRP)-coupled secondary antibodies (1:5000). After incubation, the membranes were finally assessed for expression semiquantitatively by enhanced chemiluminescence (ECL) chromogenic and gel imaging. The experiment was repeated three times.

### Griess

The accumulated level of nitrite in the cell supernatant was measured by the Griess reaction. SH-SY5Y cells (2 × 10^4^/well) were inoculated and transfected for 1 d using treatment with 1 mM MPP + for 24 h. An equal volume (50 µL) of Griess reagent (1 part 0.1% naphthaleneethylenediamine and 1 part 1% sulfonamide, 5% phosphoric acid) was added to the culture supernatant and reacted at room temperature. Nitrite levels were measured and converted using a spectrophotometer at 540 nm absorbance.

### Dual luciferase gene reporting experiment

The binding site sequences of miR-124 together with MALAT1 and MEKK3 were predicted through the StarBase database. The MEKK3-3’UTR or MALAT1-3’UTR fragment containing the miR-124 predicted binding site was cloned and inserted into the pGL3 vector to form wild-type recombinant luciferase reporter plasmids (WT-MALAT1, WT-MEKK3). The putative binding sites were mutated using a QuickChange sentinel mutagenesis kit (Stratagene, China). Mutated fragments of MALAT1 and MEKK3 were inserted into the pGL3 vector to form mutant recombinant luciferase reporter plasmids (MUT-MALAT1, MUT-MEKK3). Plasmids were extracted, and the plasmids, miR-124 mimics and NCs were transfected 48 h later.

### Animal husbandry and model building

C57BL/6J mice were purchased from the Laboratory Animal Center of Kunming Medical University. Male C57BL/6J mice weighing 20 ~ 23 g at 8 weeks old were used in the experiments and were housed in a controlled environment (21 ± 1 °C, 12 h light/dark cycle). After 7 d of acclimation, the mice were divided into normal, PD, PD + MDMA, and PD + MDMA + si-MALAT1 groups. According to the literature [18], intraperitoneal (IP) MDMA (10 mg/kg) was injected twice a day, 4 to 6 h apart, twice a week, on Tuesdays and Fridays, for 9 weeks. After the last administration of MDMA, MPTP (20 mg/kg) was injected via IP once daily for 4 days (PD + MDMA group). For the PD group, no MDMA was injected. For the PD + MDMA + si-MALAT1 group, si-MALAT1 was injected along with MDMA. Three days after the last administration, mice were anesthetized with isoflurane and underwent ice-cold PBS transcardial perfusion. The SN area was taken for subsequent testing.

#### Nissl staining

The brain samples were isolated and treated with 4% paraformaldehyde, embedded and cut. Then, the samples were stained with 0.1% methylphenol violet solution. Tissues were rinsed with running water, dehydrated with 95% ethyl alcohol, and treated with 70% ethanol. Photographs were observed using a BX51 light microscope (Olympus, Japan), and the number of stained cells in the substantia nigra pars compacta (SNpc) was counted.

### Immunohistochemical staining

Citric acid antigen repair buffer was added to sections and placed in a microwave for antigen repair, and the treated brain sections were placed in 3% H_2_O_2_ solution to inhibit endogenous peroxidase. The sections were blocked with 3% BSA at room temperature for 30 min and then incubated with the primary antibody anti-TH (1:500, Abcam, UK) in a blocking solution at 4 °C overnight. Then, the cells were incubated with secondary antibody (1:500, Abcam, UK) at room temperature for 1 h. DAB was applied, and the cells were observed under a microscope for photographs.

### TUNEL staining

Mouse brain tissue was collected and fixed by soaking it in a freshly prepared 4% paraformaldehyde solution. Then, 4 μm thick paraffin sections were prepared, which were dehydrated by gradient ethanol, cleared by xylene, and then stained by TUNEL. After adding TUNEL reaction solution, the sections were blocked and incubated for 1 h and then incubated in streptomycin-avidin-horseradish peroxidase solution for 0.5 h. In addition, hematoxylin was used to stain the nuclei. The sections were finally photographed by microscopy. The number of TUNEL-positive cells was observed.

### Statistical analysis

GraphPad 8.0 was used to analyze the data. The results are expressed as the mean ± SD. For statistical comparisons, Student’s t test was used for comparisons between two groups, and one-way ANOVA was used for comparisons among multiple groups. P < 0.05 indicates statistical significance.

## Results

### MDMA promotes the pathogenesis of PD

**Fig. 1:**
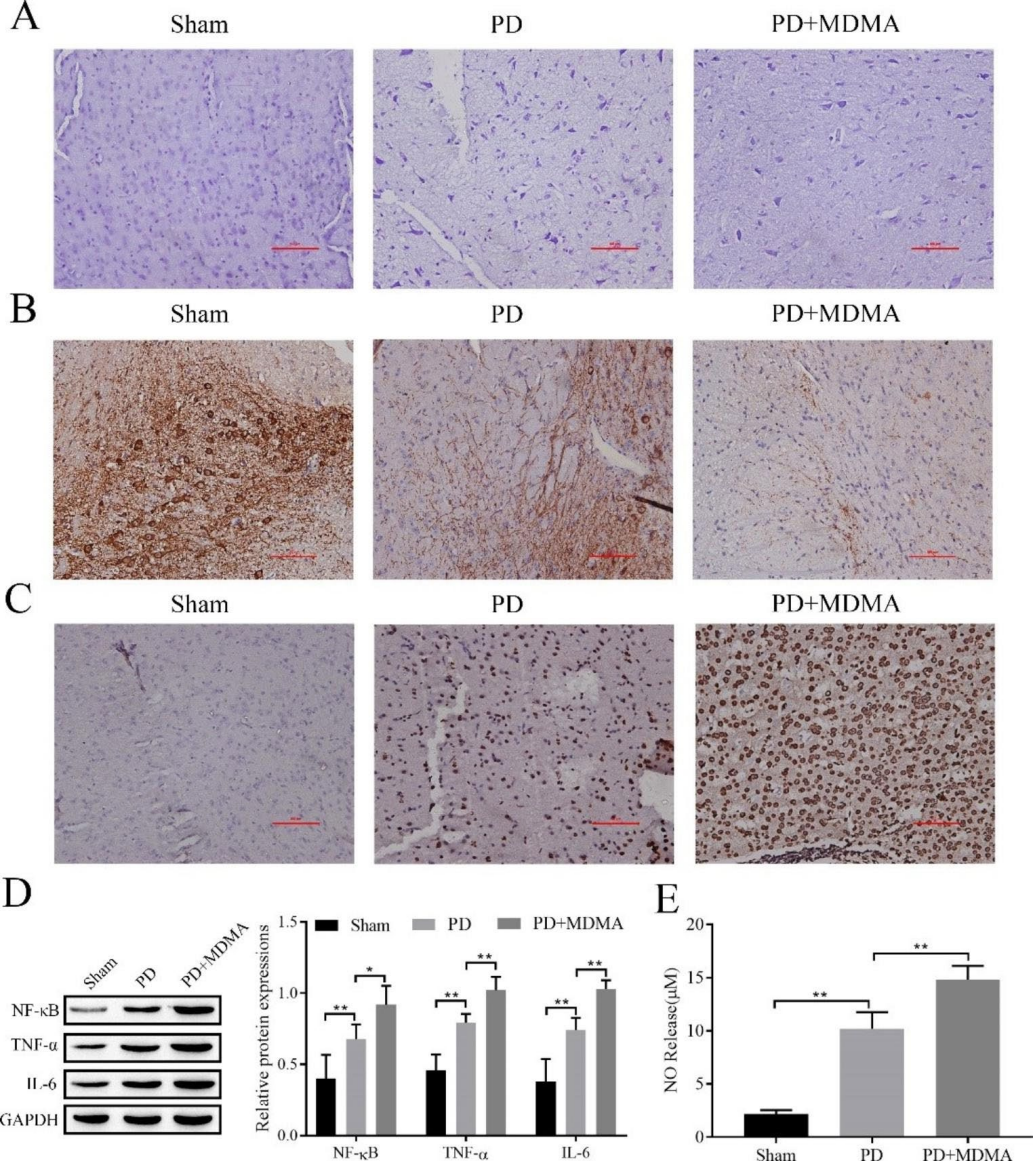
MDMA promotes the pathogenesis of PD. **A**: Nissl staining to assess neuronal damage in the SN region; **B**: Immunohistochemistry to detect TH expression in the SN; **C**: TUNEL staining to detect apoptosis in the SN region; **D**: Western blot to detect the inflammatory factors NF-κB, TNF-α, IL-6 expression; E: Griess assay NO levels. sham: sham operation control group. ^*^P < 0.05, ^**^P < 0.01

We used Nissl staining to assess neuronal damage in the SN region, and the results showed that neuronal damage in the PD group and PD + MDMA group was severe, and the latter was more severe (Fig. 1A). Tyrosine hydroxylase (TH) is widely considered an indicator of nigrostriatal PD [19]. After observing the results of immunohistochemistry, we observed TH expression in the substantia nigra (SN), and the results showed that TH expression was reduced in the SN of the PD group and PD + MDMA group, and the latter was lower (Fig. 1B). TUNEL staining was used to detect apoptosis in the SN area, and we concluded that the apoptosis rate was significantly increased in the PD group and PD + MDMA group, and the latter was more obvious (Fig. 1C). Since neuroinflammation is an important factor in promoting neuropathy in PD [20], we used Western blotting to detect the expression of the inflammatory factors NF-κB, TNF-α, and IL-6, and the results showed that their expression was significantly increased in the PD group and PD + MDMA group, and the latter was more obvious (Fig. 1D). Since NO is thought to promote the development of PD [21], we used Griess to detect NO content, and the results showed that NO content obviously increased in the PD group and PD + MDMA group, and the latter was more obvious. (Fig. 1E). It is thus clear that MDMA promotes the pathogenesis of PD.

### MDMA inhibits SH-SY5Y cell proliferation and promotes inflammation and NO release in vitro

**Fig. 2:**
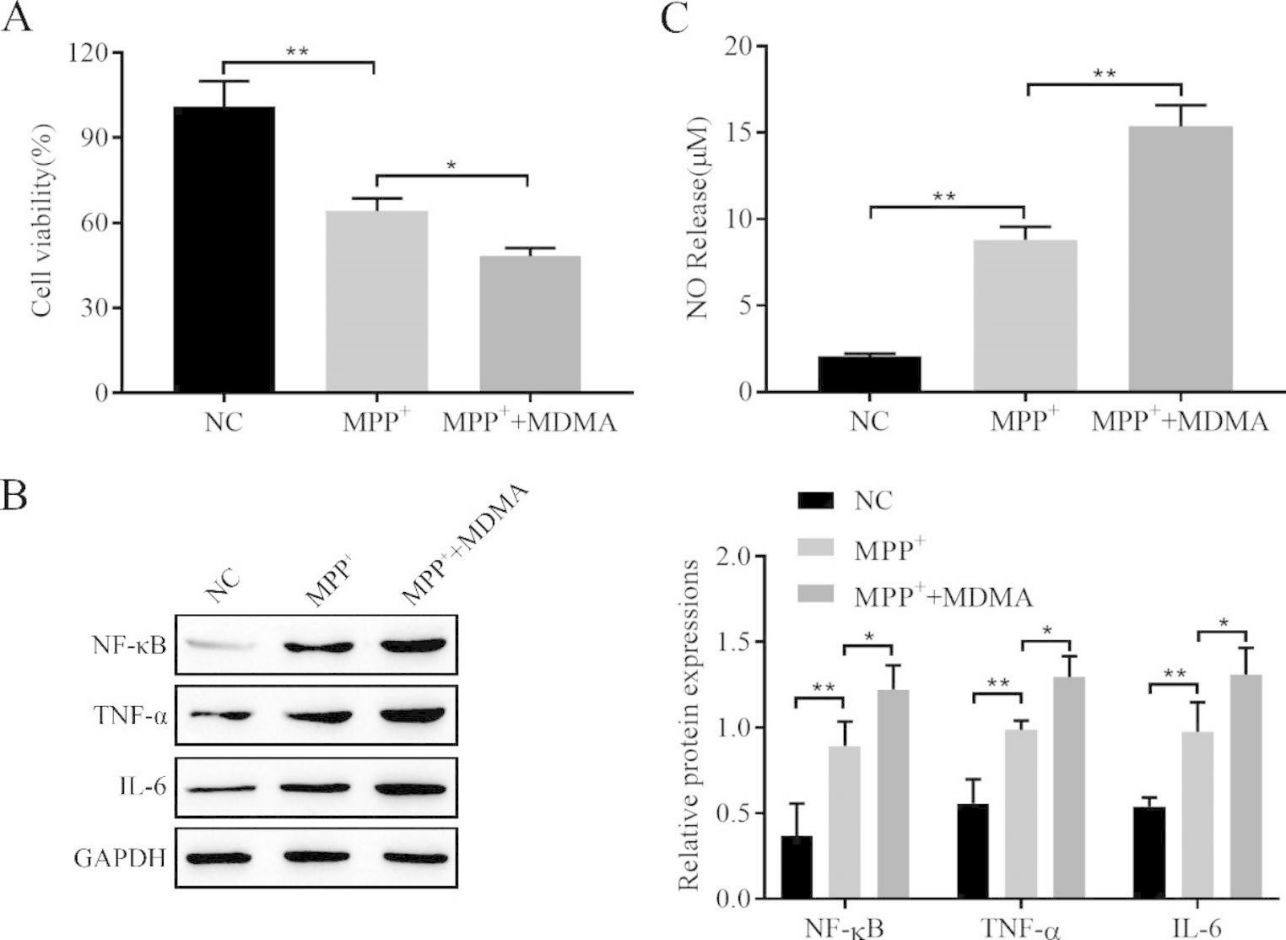
MDMA inhibits SH-SY5Y cell proliferation and promotes inflammation and NO release in vitro. **A**: CCK-8 assay for cell proliferation viability; **B**: Western blot to detect the expression of the inflammatory factors NF-κB, TNF-α, and IL-6; **C**: Griess assay to detect NO content. ^*^P < 0.05, ^**^P < 0.01

Based on the CCK-8 assay, we concluded that the proliferation viability of MPP+-treated cells was significantly reduced and further reduced by MDMA treatment (Fig. 2A). The Western blot analysis results also show that MPP + significantly upregulated NF-κB, TNF-α, and IL-6 expression and that MDMA had a stronger upward adjustment ability (Fig. 2B). The NO content was detected using Griess, and NO content increased in the MPP + group and MPP^+^+MDMA group, and the latter was more obvious (Fig. 2C). Thus, it is clear that MDMA inhibits SH-SY5Y cell proliferation and promotes inflammation and NO release in vitro.

### MDMA upregulates the expression of MALAT1

**Fig. 3:**
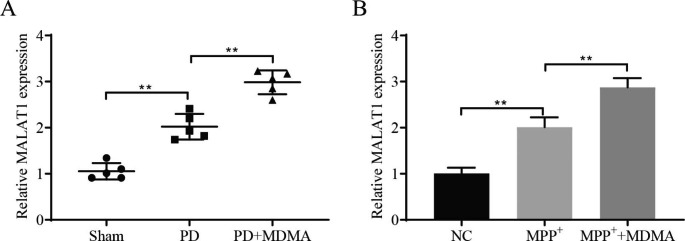
MDMA upregulates MALAT1 expression. RT‒qPCR detection of MALAT1 expression in mouse SN (**A**) and SH-SY5Y cells (**B**). ^**^P < 0.01

By analyzing the results of RT‒qPCR, we learned that the expression level of MALAT1 showed an upward trend in the PD and PD + MDMA groups, and the latter was more obvious (Fig. 3A). Similar results were obtained in cellular experiments (Fig. 3B). It is thus clear that MDMA upregulates the expression of MALAT1.

### In vitro demonstration that knockdown of MALAT1 alleviates MDMA-induced cell proliferation inhibition, inflammation and NO release

**Fig. 4:**
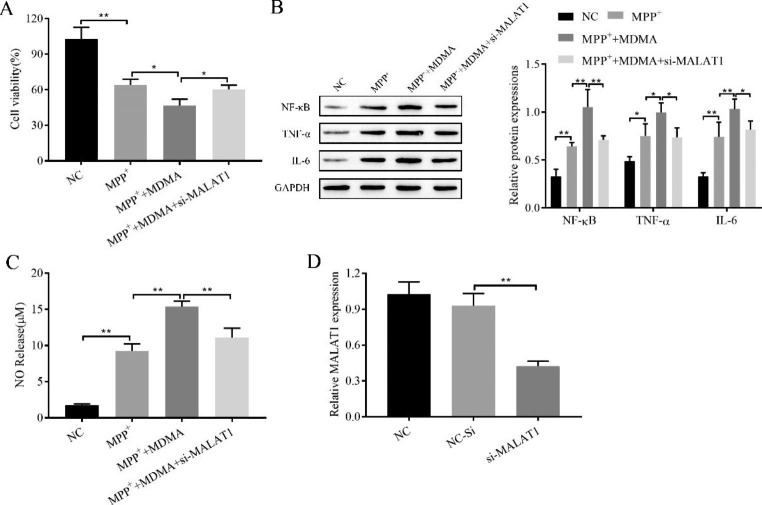
Knockdown of MALAT1 alleviates MDMA-induced cell proliferation inhibition, inflammation and NO release. **A**: CCK-8 assay for cell proliferation viability; **B**: Western blot for inflammatory factor NF-κB, TNF-α, and IL-6 expression; **C**: Griess assay for NO content; **D**: RT‒qPCR for knockdown efficiency of MALAT1. ^*^P < 0.05, ^**^P < 0.01

Furthermore, the effects of MALAT1 on MDMA-induced cell proliferation inhibition, inflammation and NO release were verified in vitro by knocking down MALAT1. Cell proliferation, viability, inflammatory factor expression and NO content were measured by CCK-8, Western blot and Griess assays, respectively. The results showed that knockdown of MALAT1 alleviated MDMA-induced cell proliferation inhibition, inflammation and NO release (Fig. 4A-C). The efficiency of MALAT1 knockdown is shown in Fig. 4D. It is thus clear that knockdown of MALAT1 alleviated the MDMA-promoting effect on PD.

### Targeted regulatory relationships between MALAT1, miR-124 and MEKK3

**Fig. 5:**
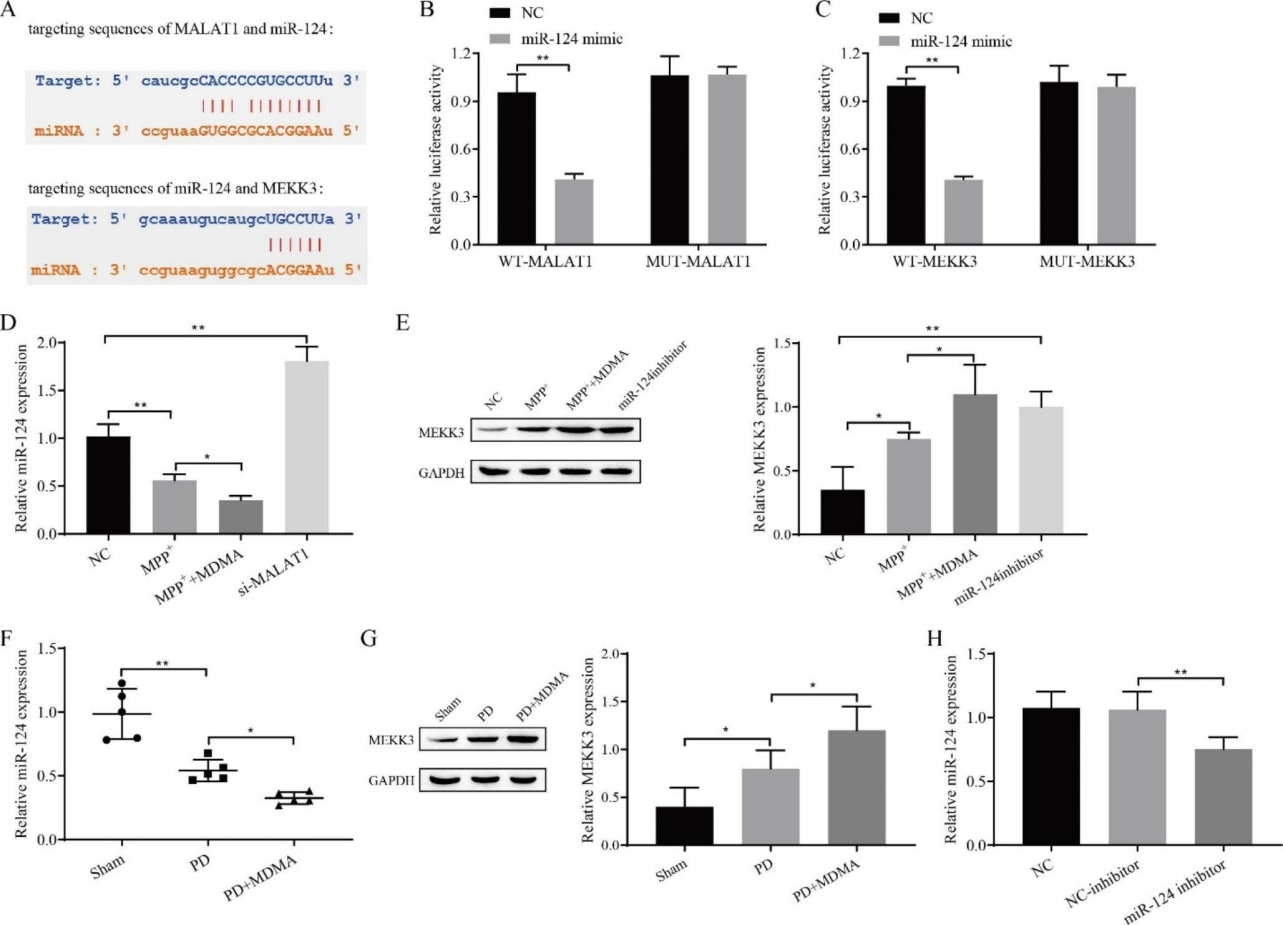
Targeted regulatory relationships between MALAT1, miR-124 and MEKK3. **A**: StarBase prediction of target binding sequences between MALAT1, miR-124 and MEKK3; **B**-**C**: Validation of the targeting relationship with dual luciferase reporter genes MALAT1 and miR-124, miR-124 and MEKK3; **D**: RT‒qPCR for miR-124 expression. E: Western blot for MEKK3 expression; F: RT‒qPCR for miR-124 expression; G: Western blot for MEKK3 expression; H: RT‒qPCR for transfection efficiency of miR-124 inhibitor. ^*^P < 0.05, ^**^P < 0.01

According to StarBase, we found a target binding sequence between MALAT1 and miR-124 and between miR-124 and MEKK3 (Fig. 5A). By analyzing the data obtained by the dual-luciferase gene reporter assay, we concluded that miR-124 significantly downregulated the luciferase activity of wild-type MALAT1 and MEKK3 vectors, while it had no significant effect in the mutant MALAT1 and MEKK3 vectors (Fig. 5B-C). After evaluation by RT‒qPCR, the quantitative data showed that the knockdown of MALAT1 significantly increased miR-124 expression. In addition, we also found that MPP + decreased the expression of miR-124, and MDMA treatment further inhibited the expression of miR-124 (Fig. 5D). In animal experiments, we also found that miR-124 expression was reduced in the PD and PD + MDMA groups, and the latter was lower (Fig. 5F). Moreover, after observing the Western blot results, we found that inhibition of both miR-124 and MPP + treatment significantly increased MEKK3 expression, and MDMA treatment further promoted the expression of MEKK3 (Fig. 5E). In animal experiments, we found that MEKK3 expression increased in the PD and PD + MDMA groups, and the increase was more obvious in the latter (Fig. 5G). The miR-124 inhibitor transfection efficiency is shown in Fig. 5H. It is thus clear that MALAT1 targets the downregulation of miR-124 expression and that miR-124 targets the downregulation of MEKK3 expression.

### MDMA inhibits cell proliferation and promotes inflammation and NO release via MALAT1/miR-124/MEKK3

**Fig. 6:**
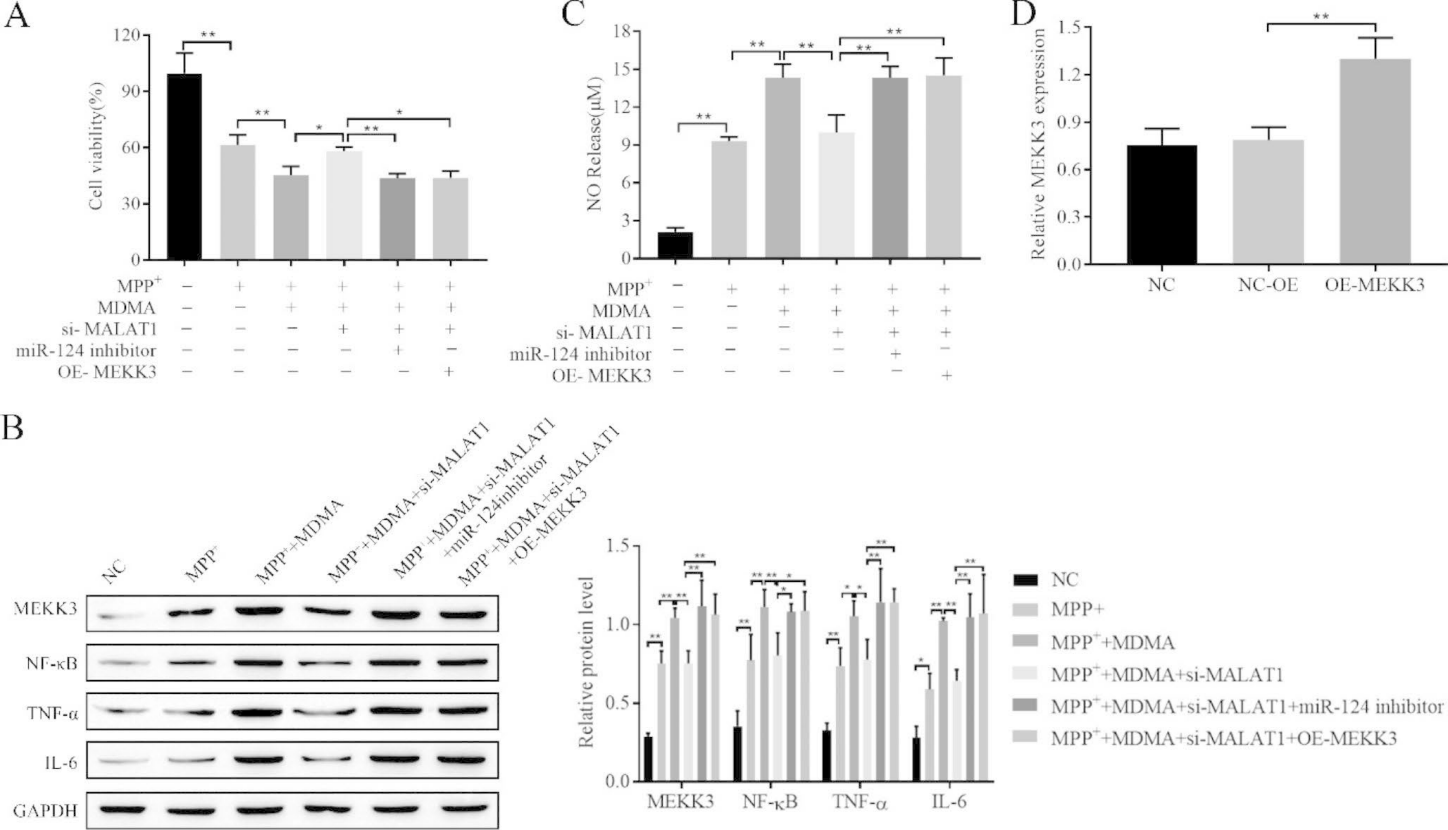
Knockdown of MALAT1 alleviates MDMA-induced cell proliferation inhibition, inflammation and NO release. **A**: CCK-8 assay for cell proliferation viability; Western blot for MEKK3 and inflammatory factors NF-κB, TNF-α, and IL-6 expression (**B**) and RT‒qPCR for OE- MEKK3 transfection efficiency (**D**); **C** : Griess assay for NO content. ^*^P < 0.05, ^**^P < 0.01

To further investigate whether MDMA affects SH-SY5Y cell proliferation, inflammation and NO release through the MALAT1/miR-124/MEKK3 molecular axis, si-MALAT1, miR-124 inhibitor, and OE-MEKK3 were transfected into SH-SY5Y cells, and CCK-8, Western blot and Griess assays were used to detect cell proliferation, MEKK3 and inflammation-related factor expression, and NO content, respectively. The results showed that inhibition of miR-124 and overexpression of MEKK3 restored the inhibitory effects of MALAT1 knockdown on MDMA-induced proliferation inhibition, MEKK3 upregulation, inflammation and promotion of NO release (Fig. 6A-C). The OE-MEKK3 transfection efficiency is shown in Fig. 6D.

### Knockout of MALAT1 attenuates the MDMA-promoting PD process via miR-124/MEKK3

**Fig. 7:**
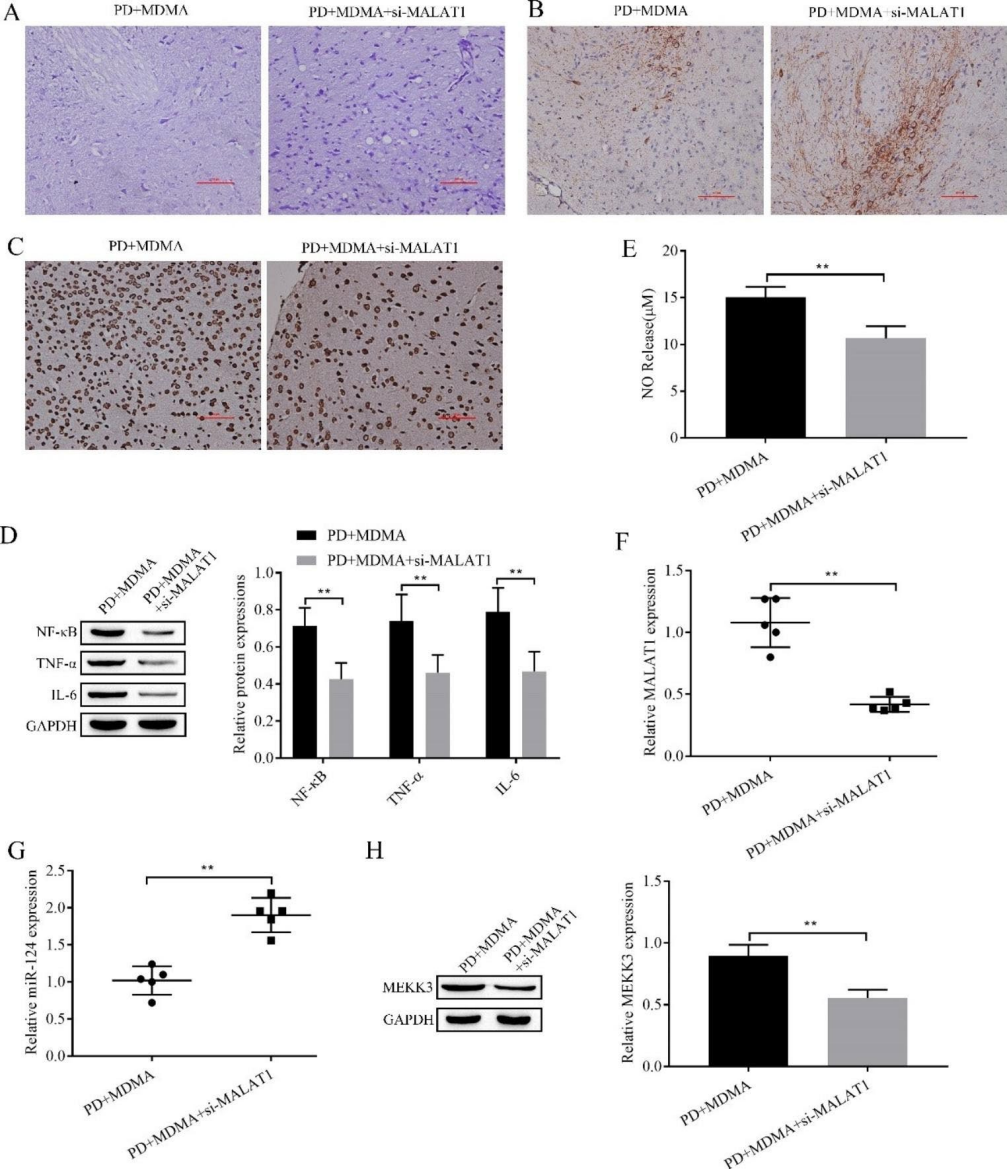
Knockdown of MALAT1 alleviates the promotion of the PD process by MDMA through miR-124/MEKK3. **A**: Nissl staining to assess neuronal damage in the SN region; **B**: Immunohistochemistry to detect TH expression in the SN; **C**: TUNEL staining to detect apoptosis in the SN region; Western blot for NF-κB, TNF-α and IL-6 expression (**D**) and MEKK3 (**H**); **E**. Griess to detect NO content; RT‒qPCR was used to detect the expression of MALAT1 (**F**) and miR-124 (**G**) . ^**^P < 0.01

After observing the results of Nissl staining, immunohistochemical staining, TUNEL staining, Western blot and Griess, we found that knockdown of MALAT1 alleviated MDMA-induced neuronal injury, TH expression inhibition, apoptosis, inflammation levels, and NO production (Fig. 7A-E). In addition, we also detected MALAT1 and miR-124 expression by RT‒qPCR, and we found that MALAT1 expression showed a clear downward trend and miR-124 expression showed a clear upward trend after knockdown of MALAT1 compared with the PD + MDMA group (Fig. 7F, G). Western blot analysis of MEKK3 expression showed that the expression of MEKK3 was significantly reduced after MALAT1 knockdown (Fig. 7H). Thus, it is clear that knockdown of MALAT1 alleviates the MDMA-promoting effect on the PD process through miR-124/MEKK3.

## Discussion

PD is a degenerative movement disorder that negatively affects daily functioning [22], and environmental factors such as drugs, chemicals or pesticides may be inextricably linked to the etiology of PD [18]. MDMA is an amphetamine-related drug that rapidly promotes the release of serotonin and dopamine (DA) in the brain, and its use in mice leads to acute hyperthermia and degeneration of striatal DA nerve endings, with neuroinflammatory and neurotoxic effects [18, 23]. However, MDMA is still used by approximately 22 million drug users worldwide [24]. The study found that MDMA enhances medical psychological assessment (MPA). Studies have found that MDMA enhances 1-methyl-4-phenyl-1,2,3,6-tetrahydropyridine (MPTP)-induced dopamine neuron degeneration and enhanced glial cell activation[18] .The early use of MDMA in macaques exacerbated MPTP-induced PD processes and associated dopaminergic damage [6]. Our study found that MDMA promoted an MPP+-induced decrease in SH-SY5Y cell viability, promoted MPP+-induced inflammation-related protein expression and NO release, promoted SN neuronal injury, inhibited TH expression, and promoted the progression of PD.

It is well known that noncoding small RNAs include a variety of RNA molecules, including miRNAs and siRNAs. They have been reported to have unique regulatory functions in a variety of diseases [25]. MALAT1 is a widely studied and highly conserved long noncoding RNA that is involved in the regulation of hyperproliferation, inflammation/immunity, and neural injury through multiple target miRNAs. Furthermore, these target miRNAs are essential in the development of various diseases, including neurological disorders [26]. Yang [27] et al. found that PD patients with high serum MALAT1 levels had lower Simple Mental State Evaluation Scale (MMSE) scores and higher serum levels of IL-1β, IL-6, TNF-α and IFN-γ. Xia [28] et al. found that lowering MALAT1 reduced MPTP-induced apoptosis and proliferation inhibition. According to the analysis of experimental data, MALAT1 has a high expression level in PD cells and animal models. MDMA further upregulated MALAT1 expression, and knockdown of MALAT1 inhibited MDMA-induced proliferation inhibition, inflammation, and NO release in SH-SY5Y cells as well as SN neuronal injury and TH expression inhibition.

miR-124 is involved in the regulation of synaptic morphology, neurotransmission and neuronal development in the nervous system [29]. Extensive preclinical evidence has demonstrated that miR-124 may interact with cell death mediators (Bim) via calpain 1/p25/cyclin-dependent kinase 5 (CDK5), nuclear factor-κB (NF-κB), signaling sensor and activator of transcription 3 (STAT3), and bcl-2. The pathway mediated by 5’ adenylate-activated protein kinase (AMPK) and extracellular signal-regulated kinase (ERK) has functions in regulating PD cell survival, neuroinflammation and other symptoms [12]. Yao [30] et al. found that miR-124 is an important factor in the development of PD. After data analysis in this experiment, we revealed that miR-124 can act as a MALAT1 target miRNA with decreased expression in PD cells and animal models. MDMA treatment further reduced miR-124 expression, and knockdown of miR-124 partially restored the proliferation inhibition, inflammation and NO release effects of knockdown of MALAT1 in MDMA-induced PD cell models.

We identified MEKK3 as a miR-124 target gene. MEKK3 is a Ser/Thr protein kinase and a member of the MAP3K family [31] that regulates various inflammatory and immune responses [32, 33]. Reviewing a large number of studies, it is known that MEKK3 has a special function in tumor development [34], cerebrovascular malformation formation [35], spinal cord ischemia/reperfusion [36] and brain development [37]. Recently, it has also been shown that MEKK3 is involved in PD regulation, and Li [38] et al. found that inhibition of MEKK3 expression inhibited proinflammatory signaling and enhanced the neuroprotective effects of PD. After data analysis in this experiment, we learned that the expression of MEKK3 showed an upregulated trend in PD cells and animal models, that MDMA treatment further upregulated MEKK3 expression and that overexpression of MEKK3 partially restored the effects of knockdown of MALAT1 on MDMA-induced proliferation inhibition, inflammation and NO release in PD cell models.

In summary, our study found that MDMA promotes MALAT1 expression and inhibits the targeted downregulation of MEKK3 by miR-124, resulting in upregulation of the expression of MEKK3 and finally jointly promoting the PD process.

### Electronic supplementary material

Below is the link to the electronic supplementary material.

Supplementary Material 1

## Data Availability

The datasets used and/or analyzed during the current study are available from the corresponding author upon reasonable request.
