# Supplementary material for: MDMA targets miR-124/MEKK3 via MALAT1 to promote Parkinson’s disease progression
Source: Mol Biol Rep. 2023 Sep 9;50(11):8889–99. doi: 10.1007/s11033-023-08775-w (PMC10635915; doi:10.1007/s11033-023-08775-w)
Supplement: Supplementary file 1 — Supplementary Material 1 [file 11033_2023_8775_MOESM1_ESM.pdf]

Editing Certificate

This document certifies that the manuscript

MDMA targets miR-124/MEKK3 via MALAT1 to promote Parkinson's disease procession

prepared by the authors

Xin Geng1, 2#, Shipeng Li1, 2#, Jinghui Li1, 2, Renli Qi1, 2,Lianmei Zhong1,2*, Hualin Yu1, 2*

was edited for proper English language, grammar, punctuation, spelling, and overall style by one or more of the highly qualified native English speaking editors at AJE.

This certificate was issued on **August 11, 2023** and may be verified on the AJE website using the verification code **FAA4-88A2-96F7-0E59-80AA**.

Neither the research content nor the authors' intentions were altered in any way during the editing process. Documents receiving this certification should be English-ready for publication; however, the author has the ability to accept or reject our suggestions and changes. To verify the final AJE edited version, please visit our verification page at aje.com/certificate. If you have any questions or concerns about this edited document, please contact AJE at support@aje.com.

AJE provides a range of editing, translation, and manuscript services for researchers and publishers around the world. For more information about our company, services, and partner discounts, please visit aje.com.
